# Numerical analysis of the porous structure of spherical activated carbons obtained from ion-exchange resins

**DOI:** 10.1038/s41598-023-50682-4

**Published:** 2024-01-02

**Authors:** Mirosław Kwiatkowski, Ping He, Valentin Valtchev

**Affiliations:** 1 Faculty of Energy and Fuels, AGH University of Krakow, al. Adama Mickiewicza 30, 30-059 Krakow, Poland; 2 Qingdao Institute of Bioenergy and Bioprocess Technology, Chinese Academy of Sciences, Qingdao, 266101 China; 3 Laboratoire Catalyse et Spectrochimie, ENSICAEN, UNICAEN, CNRS, Normandie University, 6 Marechal Juin, 14050 Caen, France

**Keywords:** Chemical physics, Chemical engineering, Physical chemistry, Computational methods, Characterization and analytical techniques, Porous materials

## Abstract

This paper presents the results of an analysis of the porous structure of spherical activated carbons obtained from cation-exchange resin beads subjected to ion exchange prior to activation. The study investigated the effects of the type of cation exchange resin, the concentration of potassium cations in the resin beads and the temperature of the activation process on the adsorption properties of the resulting spherical activated carbons. The numerical clustering-based adsorption analysis method and the quenched solid density functional theory were used to analyse the porous structure of spherical activated carbons. Based on original calculations and unique analyses, complex relationships between preparation conditions and the porous structure properties of the obtained spherical activated carbons were demonstrated. The results of the study indicated the need for simultaneous analyses using advanced methods for the analysis of porous structures, i.e., the numerical clustering-based adsorption analysis method and the quenched solid density functional theory. This approach allows a reliable and precise determination of the adsorption properties of the materials analysed, including, among other things, surface heterogeneities, and thus an appropriate selection of production conditions to obtain materials with the expected adsorption properties required for a given industrial process.

## Introduction

Porous carbonaceous adsorbents, due to their unique properties such as very high specific surface area, high hydrophobicity, extensive pore structure, and accessibility have found widespread use in various industries^1–8^. However, activated carbons in dusty form cause many technical problems, which limits their application. Therefore, there is a high demand in industry for carbonaceous materials with controlled macroscopic shapes, particularly with a spherical shape^9,10^. The properties of the raw material, and the production process methods as well as conditions, determine to a significant extent the specific surface area, pore structure, and pore size distribution^11–13^. Therefore, by selecting an appropriate precursor, appropriate production method, and controlling the production process conditions, the adsorption properties of activated carbons can be tailored to specific industrial applications^14–16^.

## Materials and methods

The paper^17^ presents the results of a study dedicated to the preparation of spherical microporous activated carbons. Cation exchange resin beads with gel-like microporous (GCB) and macroporous (MSC) structures were used as the starting material. These resin beads were prepared from DOWEX 50WX2 microporous gel (GCB) and DOWEX MSC macroporous resin (MSC).

The structure of the two types of resins used as precursors is similar. Still, their structural organisation is different, i.e., the GCB resin does not have pores larger than 3 nm, so the ions can only diffuse through the gel network and interact with ion exchange sites. In contrast, the MSC resin has large pores and an easily accessible surface, thereby facilitating ion removal^17^. However, the structure of both of these cation-exchange resins provides the opportunity to distribute the activator evenly, thus ensuring that the activation process is uniform throughout the samples^17^.

Both microporous gel-type resin beads prepared from microporous DOWEX 50WX2 gel (GCB) and macroporous DOWEX MSC resin (MSC) were subjected to ion exchange with KCl solutions of different concentrations, i.e., 0.1 M, 0.3 M, 0.5 M and 1.0 M, respectively, and were activated in an inert nitrogen atmosphere at different activation process temperatures, i.e., 700, 800, 900 and 1000 °C for an activation process duration of 2 h, with an activation temperature rise rate of 3 °C/min^17^.

In the K^+^ exchanged resin beads, K^+^ cations play the role of activator, and its activation efficiency is similar to that of the conventionally used KOH, which leads to oxidation of carbon and decomposition of the hydrocarbons into H_2_, CO, CO_2_ and H_2_O, according to the reaction below:

$${\text{6K}}^{ + } + {\text{2C}} + {\text{2OH}}^{ - } \to {\text{2K}} + {\text{2K}}_{{2}} {\text{CO}}_{{3}} + {\text{H}}_{{2}}$$

$${\text{K}}_{{2}} {\text{CO}}_{{3}} \to {\text{K}}_{{2}} {\text{O}} + {\text{CO}}_{{2}}$$

$${\text{K}}_{{2}} {\text{CO}}_{{3}} + {\text{2C}} \to {\text{2K}} + {\text{3CO}}$$

$${\text{K}}_{{2}} {\text{O}} + {\text{C}} \to {\text{2K}} + {\text{CO}}$$

Spherical activated carbons prepared from DOWEX 50WX2 microporous gel (GCB) and DOWEX MSC macroporous resin (MSC) were designated GCB-m-t and MSC-m-t, respectively, where m is the concentration of the KCl solution (in mol/dm^3^) and t is the temperature of the activation process in °C^17^.

Among other studies, the physicochemical properties of the obtained spherical activated carbons were investigated to assess the influence of the type of raw material, the potassium chloride concentration, and the temperature of the activation process on the adsorption properties of the obtained adsorbents^17^. Nitrogen adsorption isotherms were determined for the resulting spherical activated carbons using a volumetric method (Micromeritics ASAP 2460) at 77 K. The samples were outgassed at 300 °C for 12 h under vacuum conditions to remove the adsorbed water, gas and impurities^17^.

Based on the adsorption isotherms obtained, the specific surface area of the resulting spherical adsorbents was determined using the Brunauer—Emmett—Teller (BET) method in the relative pressure range *P*/*P*_*0*_ from 0.05 to 0.25^18^. In turn, the total pore volume *V*_*total*_ was calculated from the amount of nitrogen adsorbed at a relative pressure *P*/*P*_*0*_ of approximately 0.99, and the micropore volume *V*_*micro*_ was determined using the t-plot method^19^. Pore size distributions were determined using a method based on Barrett-Joyner-Halenda (BJH) theory^20^.

In the microporous active carbons adsorption process is much more intensive in micropores with highly increased adsorption potential than in larger pores. Hence, adsorption capacity and energy distribution are linked with the geometrical properties of pores. To successfully synthesize and apply novel carbonaceous adsorbents, their surface, and structural properties must be precisely and reliably characterized.

The Brunauer–Emmett–Teller (BET) and t-plot methods, which are commonly used in the analysis of porous structures, as well as the Barrett-Joyner-Halenda (BJH) method, which is used less and less frequently to determine pore size distributions, have been criticised for oversimplifying assumptions that are far removed from reality and, among other things, do not take surface heterogeneities into account, as well as underestimating pore sizes^21^.

Due to the increasing demands placed on adsorbents, technologies for their manufacture are being improved, which, however, requires a precise assessment of the porous structure taking into account surface heterogeneities, which is not fully provided by the BET, t—plot, and BJH methods used in previous studies^17^. Therefore, a concept was developed to analyse the adsorption process and porous structure of spherical activated carbons using advanced analysis methods taking into account, among other things, surface heterogeneity and pore geometry, i.e., the numerical clustering-based adsorption analysis method (LBET)^22–24^ and the quenched solid density functional theory method (QSDFT)^26,26^.

The LBET method, the theoretical foundations for the LBET models and their derivation, and the numerical fast multivariate procedure of adsorption system identification, were described in detail in earlier publications^22–24^. The LBET models have five adjusted parameters: *V*_*hA*_ [cm^3^/g], *Q*_*A*_ [J/mol]_,_* α*, *β* and *B*_*C,*_ which can be adjusted by fitting LBET equation to the adsorption isotherm, with a chosen variant of the surface energy distribution function [38–40]. Additionally, a fast multivariate method of fitting the LBET models to the adsorption isotherms was employed to determine the value of the surface heterogeneity parameter *h* and the shape of adsorption energy distribution on the first layer.

The problems associated with traditional methods used to determine pore size distributions, such as BJH and DFT, have been solved by using a method based on non-local density functional theory (NLDFT)^25^. The NLDFT method allows accurate pore size information to be obtained from both the adsorption and desorption branches of the hysteresis loop, which is crucial for the pore size characterisation of complex pore networks. However, one disadvantage of the NLDFT method is that the surface of the solid is treated as chemically homogeneous and molecularly smooth. This leads to different layering steps in the theoretical adsorption isotherms, which are not observed experimentally, resulting in artifacts in the pore size distribution of NLDFTs with a pore size of around 1 nm^21^. In order to account for the effect of surface heterogeneities occurring in carbon materials, a method (QSDFT) was developed that takes into account surface heterogeneities through a roughness parameter representing surface corrugations at the molecular level^26^.

## Discussion of the obtained results

**Figure 1:**
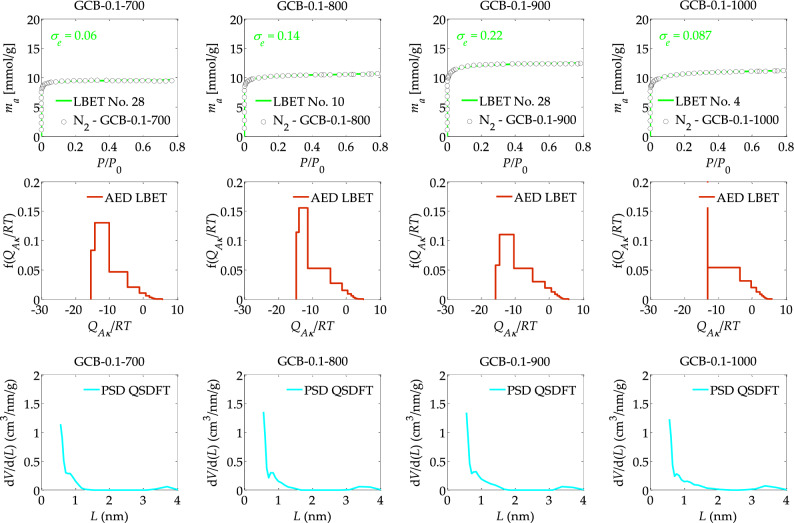
The nitrogen adsorption isotherms and the results of the identification of the adsorption systems via the LBET method and adsorption energy distributions (AED) obtained for the spherical activated carbons prepared at different temperatures of the activation process, i.e., 700, 800, 900 and 1000 °C.

**Figure 2:**
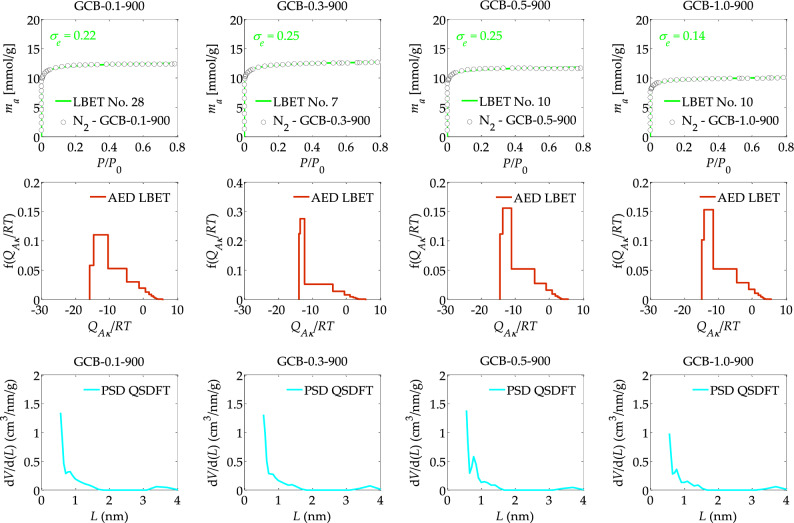
The nitrogen adsorption isotherms and the results of the identification of the adsorption systems via the LBET method, and adsorption energy distributions (AED) obtained for the spherical activated carbons prepared from DOWEX 50WX2 microporous gel (GCB) at different solution concentration, i.e., 0.1, 0.3, 0.5 and 1.0 M KCl used for ion exchange.

**Figure 3:**
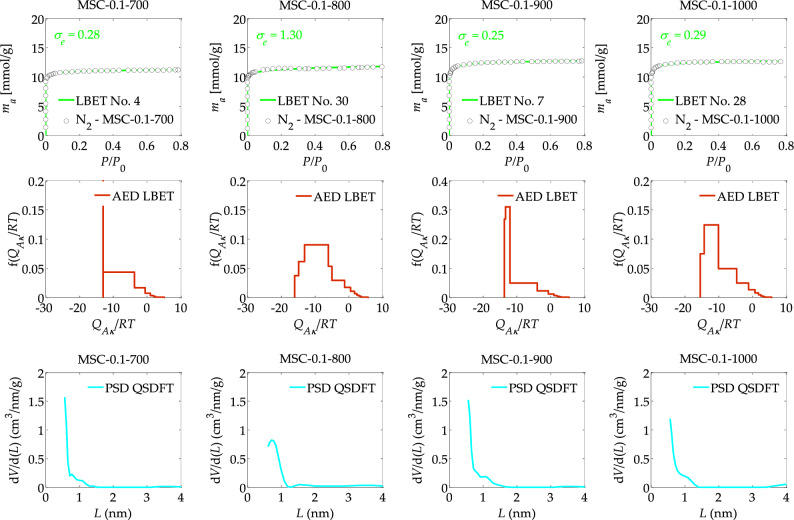
The nitrogen adsorption isotherms and the results of the identification of the adsorption systems via the LBET method, and adsorption energy distributions (AED) obtained for the spherical activated carbons prepared from DOWEX MSC macroporous resin (MSC) ion-exchanged with 0.1 M KCl solution at different activation process temperatures, i.e. 700, 800, 900 and 1000 °C.

**Figure 4:**
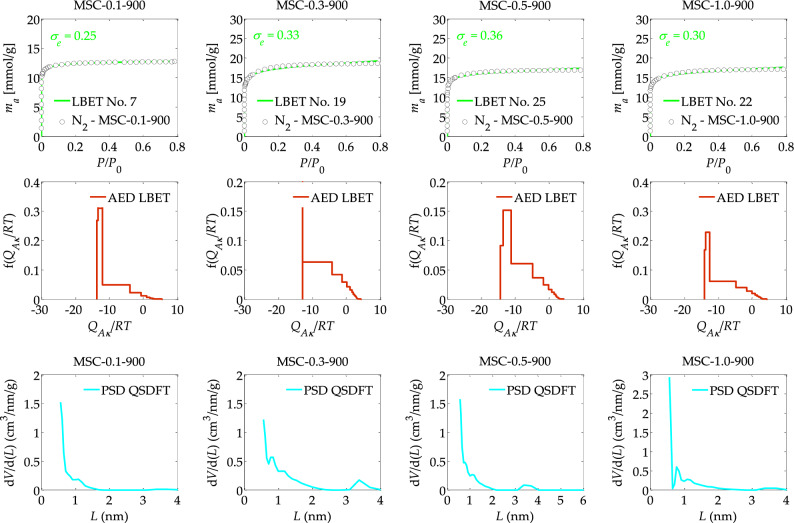
The nitrogen adsorption isotherms and the results of the identification of the adsorption systems via the LBET method and adsorption energy distributions (AED) obtained for the spherical activated carbons prepared from DOWEX MSC macroporous resin (MSC) at different solution concentration, i.e., 0.1, 0.3, 0.5 and 1.0 M KCl used for ion exchange, subjected to carbonisation at 900 °C.

The results of the analyses carried out using the LBET and QSDFT methods are summarised in Tables 1, 2, 3, 4 and shown in Figs. 1, 2, 3, 4. In the first part of the study, the structure analysis of spherical activated carbons obtained from microporous gel-type resin beads prepared from DOWEX 50WX2 microporous gel (GCB) and subjected to ion exchange with 0.1 M KCl solution at different temperatures of the activation process, i.e., 700, 800, 900 and 1000 °C, was performed on the basis of nitrogen adsorption isotherms. The results of the analyses performed via the LBET and QSDFT methods are presented in Table 1 and Fig. 1. Based on the results obtained with the LBET method, it can be observed that, in the case of sample GCB-0.1-700 subjected to ion exchange with a 0.1 M KCl solution and an activation process at 700 °C, the type of the best-fitted LBET model indicates the presence of geometric growth limitations of clusters, related to the presence of narrow micropores.

**Table 1:** Parameters characterizing the porous structure of the activated carbons obtained from microporous gel-type resin beads prepared from DOWEX 50WX2 microporous gel (GCB) and subjected to ion exchange with 0.1 M KCl solution at different temperatures of the activation process, i.e., 700, 800, 900 and 1000  °C, based on the analysis of N_2_ adsorption isotherms using the LBET and QSDFT methods.

Material	*Model No*	*h*	*V*_*hA*_ [cm^3^/g]	*α*	*β*	*Q*_*A*_**/***RT*	*B*_*C*_	*σ*_*e*_	*w*_*id*_	*S*_*QSDFT*_ [m^2^/g]	*V*_*QSDFT*_ [cm^3^/g]
GCB-0.1–700	28	9	0.303	0.55	1.00	− 15.43	34.56	0.18	0.62	1011	0.333
GCB-0.1–800	10	7	0.332	0.62	1.00	− 14.65	17.97	0.14	0.25	1073	0.365
GCB-0.1–900	28	9	0.396	0.68	1.00	− 15.79	35.10	0.22	0.26	1246	0.425
GCB-0.1–1000	4	3	0.360	0.67	1.00	− 13.11	38.09	0.087	0.46	1050	0.386

Where: *V*_*hA*_: the volume of the first adsorbed layer, *Q*_*A*_/*RT*: the dimensionless energy parameter for the first adsorbed layer; *B*_*C*_: the dimensionless energy parameter for the higher adsorbed layers; *α*: the geometrical parameter of the porous structure determining the height of the adsorbate molecule clusters; *β*: the geometrical parameter of the porous structure determining the width of the adsorbate molecule clusters; *h*: the surface heterogeneity parameter; S_*QSDFT*_: the total surface area, *V*_*QSDFT*_: the volume of micropores.

**Table 2:** Parameters characterizing the porous structure of the activated carbons obtained from microporous gel-type resin beads prepared from DOWEX 50WX2 microporous gel (GCB) at different solution concentration, i.e., 0.1, 0.3, 0.5 and 1.0 M KCl used for ion exchange, of GCB samples subjected to carbonisation at 900 °C, based on the analysis of N_2_ adsorption isotherms using the LBET and DFT methods.

Material	*Model No*	*h*	*V*_*hA*_ [cm^3^/g]	*α*	*β*	*Q*_*A*_**/***RT*	*B*_*C*_	*Z*_*A*_	*σ*_*e*_	*w*_*id*_	*S*_*QSDFT*_ [m^2^/g]	*V*_*QSDFT*_ [cm^3^/g]
GCB-0.1–900	28	9	0.396	0.68	1.00	15.79	35.10	0.633	0.22	0.16	1246	0.425
GCB-0.3–900	7	5	0.404	0.63	1.00	13.90	34.70	0.582	0.25	0.21	1285	0.455
GCB-0.5–900	10	7	0.374	0.62	1.00	14.40	29.15	0.595	0.2	0.27	1162	0.401
GCB-1.0–900	10	7	0.319	0.65	1.00	14.82	29.72	0.607	0.14	0.19	1023	0.355

**Table 3:** Parameters characterizing the porous structure of the activated carbons obtained from DOWEX MSC macroporous resin (MSC) ion-exchanged with 0.1 M KCl solution at different activation process temperatures, i.e., 700, 800, 900 and 1000 °C, based on the analysis of N_2_ adsorption isotherms using the LBET and DFT methods.

Material	*Model No*	*h*	*V*_*hA*_ [cm^3^/g]	*α*	*β*	*Q*_*A*_**/***RT*	*B*_*C*_	*σ*_*e*_	*w*_*id*_	*S*_*QSDFT*_ [m^2^/g]	*V*_*QSDFT*_ [cm^3^/g]
MSC-0.1–700	4	3	0.354	0.47	1.00	− 12.98	22.91	0.28	0.18	1235	0.369
MSC-0.1–800	30	9	0.382	0.63	1.00	− 15.99	34.50	1.3	0.22	1272	0.447
MSC-0.1–900	7	5	0.404	0.56	1.00	− 13.67	28.70	0.25	0.32	1311	0.417
MSC-0.1–1000	28	9	0.407	0.60	1.00	− 15.40	35.10	0.29	0.59	1346	0.529

**Table 4:** Parameters characterizing the porous structure of the activated carbons from DOWEX MSC macroporous resin (MSC) at different solution concentrations, i.e., 0.1, 0.3, 0.5 and 1.0 M KCl used for ion exchange, subjected to carbonisation at 900 °C, based on the analysis of N_2_ adsorption isotherms using the LBET and DFT methods.

Material	*Model No*	*h*	*V*_*hA*_ [cm^3^/g]	*α*	*β*	*Q*_*A*_**/***RT*	*B*_*C*_	*σ*_*e*_	*w*_*id*_	*S*_*QSDFT*_ [m^2^/g]	*V*_*QSDFT*_ [cm^3^/g]
MSC-0.1–900	7	5	0.404	0.56	1.00	− 13.67	28.70	0.25	0.32	1311	0.417
MSC-0.3–900	19	3	0.580	0.75	1.00	− 12.80	8.14	0.33	0.54	1592	0.642
MSC-0.5–900	25	7	0.520	0.71	1.00	− 14.30	7.93	0.36	0.47	1560	0.580
MSC-1.0–900	22	5	0.526	0.74	1.00	− 13.98	7.78	0.3	0.41	1502	0.569

The aforementioned sample GCB-0.1-700 is characterised by a strongly heterogeneous surface, as indicated by the value of the surface heterogeneity parameter *h* (*h* = 9). This sample is also characterised by a large value for the volume of the first adsorbed layer (*V*_*hA*_ = 0.303 cm^3^/g) and a significant total surface area (*S*_*QSDFT*_ = 1011 m^2^/g) and micropores volume (*V*_*QSDFT*_ = 0.333 cm^3^/g), clearly indicating a significantly developed micropore structure. The LBET analysis also showed that in the pores of spherical activated carbon GCB-0.1–700 medium-height, non-branching clusters of adsorbate molecules are formed, as indicated by the values of the geometrical parameters *α*, and *β*, i.e., the height and width of the clusters of adsorbate molecules, respectively (*α* = 0.55, and *β* = 1.00). The values of the energy parameters successively for the first adsorbed layer *Q*_*A*_*/RT* and the subsequent layers *B*_*C*_ (*Q*_*A*_*/RT* = −15.43, and *B*_*C*_ = 34.56) indicate the existence of preferential conditions for the occurrence of single-layer and multilayer adsorption processes with a small number of adsorbed layers. Note that the pore volume value calculated by the QSDFT method is close to the volume value of the first adsorbed layer *V*_*hA*_ calculated by the LBET method, indicating the dominant contribution of micropores to the total pore volume.

The fit of the model isotherm to the empirical data N_2_-GCB-0.1-700, as indicated by the dispersion of the fit error *σ*_e_ is very good (*σ*_e_ = 0.18), as is the identifiability of the adsorption system (*w*_*id*_ = 0.62), which guarantees the high reliability and validity of the results obtained.

The shape of the AED adsorption energy distribution on the surface of sample GCB-0.1–700 determined by the LBET method indicates a predominant contribution of high-energy sites with a narrow energy range and a significant contribution of adsorption sites with a wide range of sites and lower adsorption energy (see Fig. 1). The shape of the pore size distribution of the PSD indicates a significant development of the structure of the smallest micropores below 1 nm, especially micropores with sizes smaller than 0.5 nm. Also of note is the small peak in the range of smaller mesopores indicating the presence of small mesopores in the pore structure of sample GCB-0.1-700. The next spherical activated carbon sample analysed was the one labelled GCB-0.1-800, i.e., obtained from GCB cation-exchange resin subjected to ion exchange with a 0.1 M KCl solution and a carbonisation process at 800 °C.

The number of the best-fit LBET model indicates, in contrast, to sample GCB-0.1-700, limitations in the expansion of clusters of adsorbate molecules related to the competitive expansion of neighbouring clusters of nitrogen molecules. The surface of sample GCB-0.1-800 is characterised by a lower degree of heterogeneity, i.e., *h* = 7, compared to sample GCB-0.1-700 and a slightly higher value for the parameters: volume of the first adsorbed layer *V*_*hA*_, total surface area S_*QSDFT*_ and micropores volume *V*_*QSDFT*_, as well as the parameter *α*, indicating a slightly higher development of its porous structure. However, sample GCB-0.1-800 is characterised by a practically two times lower value of the dimensionless energy parameter for the higher layers (*B*_*C*_ = 17.97) compared to sample GCB-0.1-700 for which *B*_*C*_ = 34.56, indicating significantly worse energy conditions for the occurrence of the multilayer adsorption process.

The fit of the model isotherm to the empirical isotherm N_2_-GCB-0.1-800 is very good, but the significantly lower value of the identifiability coefficient of the *w*_*id*_ adsorption system (*w*_*id*_ = 0.25), compared to the previously analysed sample GCB-0.1-700, is noteworthy. This may indicate that there is some deviation of the actual pore structure from the model structure implemented in the LBET method. The values of total surface area *S*_*QSDFT*_ and micropores volume *V*_*QSDFT*_ calculated by the QSDFT method for sample GCB-0.1-800 are noticeably larger compared to those determined for sample GCB-0.1-700, obtained at a lower carbonisation temperature. The shape of the distribution of AED adsorption energy values on the surface of spherical activated carbon GCB-0.1-800 indicates a higher adsorption energy in the smallest pores and a narrower range of values, while the shape of the pore size distribution indicates a minimally higher proportion of micropores in the range of approximately 0.5 nm to 1.2 nm in the total porosity in the analysed sample compared to the sample obtained at a lower activation temperature (see Fig. 1).

The next adsorbent analysed was spherical activated carbon designated GCB-0.1-900, i.e., obtained at an activation temperature of 900 °C. This material was characterised by the greatest development of the microporous structure among all analysed samples obtained from GCB cation exchange resin subjected to ion exchange with 0.1 M KCl solution, as indicated by the values of the parameters *V*_*hA*_, *α*, *S*_*QSDFT*_, *V*_*QSDFT*_, i.e., *V*_*hA*_ = 0.396 cm^3^/g, *α* = 0.68, *S*_*QSDFT*_ = 1246 m^2^/g, *V*_*QSDFT*_ = 0.425 cm^3^/g, respectively.

The shape of the adsorption energy distribution determined for sample GCB-0.1-900 is very similar to the analogous distribution obtained for sample GCB-0.1-700. In contrast, the shape of the PSD pore size distribution indicates a higher proportion of larger micropores in the total porosity of spherical activated carbon GCB-0.1-900. However, the next spherical activated carbon sample analysed, obtained at a carbonisation temperature of 1000 °C designated GCB-0.1-1000, was already characterised by lower values of the parameters *V*_*hA*_, *α*, *S*_*QSDFT*_, *V*_*QSDFT*_ (*V*_*hA*_ = 0.360 cm^3^/g, *α* = 0.67, *S*_*QSDFT*_ = 1050 m^2^/g, *V*_*QSDFT*_ = 0.386 cm^3^/g) compared to the values of these parameters determined for sample GCB-0.1-900. However, in contrast to the previously analysed samples, the surface of this GCB-0.1-1000 sample was characterised by the smallest degree of surface heterogeneity, i.e., *h* = 3, which may be of great practical importance. The shape of the adsorption energy distribution on the surface of spherical activated carbon GCB-0.1-1000, indicates the presence of a predominant proportion of sites of equal energy, indicating the significant presence of micropores comparable in size to a nitrogen molecule.

Based on studies and analyses devoted to assessing the influence of the activation process temperature, it can be concluded that in the case of spherical activated carbons obtained from cation-exchange resin subjected to ion exchange with 0.1 M KCl solution, the optimum temperature for the activation process is 900 °C, which ensures the best adsorption properties of the material. However, if the priority is to obtain a material with as little surface heterogeneity as possible and a significant proportion of micropores comparable in size to the nitrogen molecule, then an activation temperature of 1000 °C provides the right conditions for this.

The study also analysed the effect of solution concentration, i.e., 0.1, 0.3, 0.5, and 1.0 M KCl used for ion exchange, of GCB samples subjected to carbonisation at 900 °C and the results of calculations and analyses performed using LBET and QSDFT methods are presented in Table 2 and Fig. 2. Of the analysed spherical GCB activated carbons obtained at 900 °C, the samples obtained at KCl solution concentrations of 0.1 and 0.3 M were characterised by the greatest development of the porous structure as indicated by the values of the parameters *V*_*hA*_, *α*, *S*_*QSDFT*_ and *V*_*QSDFT*_ (see Table 2). Sample GCB-0.1-900 exhibits the highest degree of surface heterogeneity, i.e., *h* = 9, and sample GCB-0.3-900 having the lowest, i.e., *h* = 7.

The energy distribution shapes shown in Fig. 2 for spherical GCB activated carbon samples obtained at different KCl solution concentrations and at a carbonisation temperature of 900 °C, indicate a wide energy range of primary adsorption sites, with a significant proportion of high-energy sites on the surface of said samples. On the other hand, the shapes of the pore size distributions PSD obtained for spherical activated carbons obtained at an activation temperature of 900 °C indicate a significant development of the structure of micropores with sizes below 0.5 nm and a significant proportion of larger micropores in the range from about 0.5 nm to 1.0 nm, as well as a proportion of larger micropores with sizes greater than 1.0 nm. Also noticeable on the determined pore size distributions is the contribution of small mesopores to the total porosity.

In the second part of the study, spherical activated carbons obtained from DOWEX MSC macroporous resin (MSC) were analysed. As in the first part of the study, the influence of KCl solution concentration and carbonisation temperature on the porous structure parameters and adsorption properties of the obtained spherical activated carbons was considered.

The results of the analysis of the effect of the carbonisation temperature on the adsorption properties of spherical activated carbons obtained from DOWEX MSC macroporous resin (MSC) ion-exchanged with 0.1 M KCl solution at different activation process temperatures, i.e., 700, 800, 900 and 1000 °C, are summarised in Table 3 and Fig. 3.

From the results collected in the aforementioned Table 3, it can be observed that the sample designated MSC-0.1-700, i.e., obtained from the macroporous DOWEX MSC resin, subjected to ion exchange with a KCl solution of 0.1 M, at a carbonisation temperature of 700 °C, is characterised by the lowest degree of surface heterogeneity (*h* = 3) of all analysed MSC samples obtained from DOWEX MSC macroporous resin, subjected to ion exchange with KCl solution of 0.1 M.

The MSC-0.1-700 sample is also characterised by the smallest development of the porous structure, as indicated by the values of the parameters of the volume of the first adsorbed layer *V*_*hA*_ (*V*_*hA*_ = 0.354 cm^3^/g), the height of clusters of adsorbate particles *α* (*α* = 0.47) and the total surface area *S*_*QSDFT*_ (*S*_*QSDFT*_ = 1235 m^2^/g) and micropores volume *V*_*QSDFT*_ (*V*_*QSDFT*_ = 0.369 cm^3^/g). Also notable are the lowest values of the energy parameters for the first adsorbed layer (*Q*_*A*_*/RT*) as well as the higher layers, respectively, of all the samples analysed (*Q*_*A*_*/RT* = −12.98, *B*_*C*_ = 22.91).

As the carbonisation temperature increases, there is a successive development of the porous structure of spherical activated carbons obtained from the macroporous DOWEX MSC resin, subjected to ion exchange with a KCl solution of 0.1 M. For the sample labelled MSC-0.1-1000, and therefore obtained from a temperature of 1000 °C, the highest values of the parameters *V*_*hA*_, *α*, *S*_*QSDFT*_, *V*_*QSDFT*_ were obtained i.e. *V*_*hA*_ = 0.407 cm^3^/g, *α* = 0.60, *S*_*QSDFT*_ = 1346 m^2^/g, *V*_*QSDFT*_ = 0.529 m^2^/g, indicating the greatest development of the porous structure.

The shapes of the adsorption energy distributions on the surface of the analysed samples shown in Fig. 3 confirm the previous observations. Namely for the sample MSC-0.1-700, a distribution with a dominant share of high-energy adsorption sites and a share of sites with varying energy was obtained, while for the other MSC samples there is already a larger energy spectrum of adsorption sites. On the other hand, the shapes of the PSD pore size distributions determined for spherical MSC activated carbons, indicate the predominant contribution of micropores to the structure of these materials; however, the shape of the PSD determined for the MSC-0.1-800 sample, which differs from the other samples analysed, is noteworthy, indicating a wider energy range of primary adsorption sites and thus greater surface energy heterogeneity.

Another aspect considered as part of the ongoing research was the effect of the concentration of the KCl solution used for cation exchange (i.e., 0.1, 0.3, 0.5, 1.0 M), on the formation of the porous structure of spherical activated carbons obtained from macroporous DOWEX MSC resin at a carbonisation temperature of 900 °C, and the results of the analyses are presented in Table 4. These results show that the material with the smallest structure development was obtained at a KCl solution concentration of 0.1 M, i.e., spherical activated carbon designated MSC-0.1-900.

On the other hand, the sample obtained at a KCl solution concentration of 0.3 M, i.e., MSC-0.3-900 was characterised by the most developed microporous structure as indicated by the highest values of the parameters *V*_*hA*_, *α*, *S*_*QSDFT*_, *V*_*QSDFT*_, i.e., *V*_*hA*_ = 0.580 cm^3^/g, *α* = 0.75, *S*_*QSDFT*_ = 1311 m^2^/g, *V*_*QSDFT*_ = 0.417 cm^3^/g and the lowest degree of surface heterogeneity as indicated by the value of the parameter *h* (*h* = 3).

Further increasing the concentration of the KCl solution resulted in minimal destruction of the porous structure, as indicated by a decrease in the values of the parameters *V*_*hA*_, *α*, S_*QSDFT*_, *V*_*QSDFT*_, observed successively for the samples MSC-0.5-900 and MSC-1.0-900, respectively.

Analysis of the adsorption energy distributions on the first layer, shown in Fig. 4, indicates that there is a wide energy spectrum of primary adsorption sites in the samples studied, with the sample obtained at a KCl solution concentration of 0.3 M being characterised by a predominant proportion of adsorption sites with equal adsorption energy, i.e., pores that are most likely to contain one adsorbed nitrogen molecule.

Analysis of PSD plots determined for samples obtained from the macroporous DOWEX MSC resin at a carbonisation temperature of 900 °C, at different KCl concentrations, generally indicates a significant proportion of micropores with sizes smaller than approximately 0.6 nm and the presence of larger micropores in the structure of the adsorbents analysed.

## Conclusions

This article presents the results of a study devoted to the analysis of the porous structure of spherical microporous activated carbons obtained from cation exchange resin beads with gel-like microporous structures (DOWEX 50WX2) and macroporous resin (DOWEX MSC). The analyses carried out showed a significant influence of the preparation conditions, i.e., the concentration of the KCl solution used for cation exchange, on the structure of the obtained spherical activated carbons and the temperature of the activation process. With the increase in the activation temperature, the oxidation of carbon and decomposition of the hydrocarbons into H_2_, CO, CO_2_ and H_2_O gradually intense. The generated gases diffuse within the carbon matrix and contribute to porosity development. Besides, chemical and thermal activation happen simultaneously, maximizing the efficiency of the reaction and the development of porosity. Therefore, the increase in the activation temperature causes the increase in the specific surface area and pore volume of the prepared materials. In turn increase of the concentration of the KCl solution results in an increased K^+^ content in resin precursor. Then, the exchanged K^+^ cations within resin play the role of activator in carbonization step, and too high the K^+^ content in resin precursor results in the violence of the reaction occurring in the volume of resin beads, which can caused as a consequence the destruction of the porous structure of the materials. In the case of the GCB resin, it has no discrete pores, only solute ions could diffuse through the gel network. Different to GCB resin beads, MSC resin exhibits large pores and a highly accessible surface. So, different structural organizations will lead to different diffusion paths during the carbonization process and thus carbon beads with different pore structures will be obtained. The presence of large well-defined pores in MSC resin can ensure the efficient release of generated gases during the carbonization process, which retained morphology of the carbon beads and achieved larger surface areas and pore volumes.

As demonstrated in the study, the relationships between the preparation conditions and the porous structure properties of the obtained spherical activated carbons are complex. Therefore, only the simultaneous application of advanced methods of porous structure analysis, such as LBET and QSDFT methods, allows a reliable determination of the adsorption properties of these materials and, thus, the optimal selection of preparation conditions to obtain an adsorbent with the specific physicochemical properties expected in a given adsorption industrial process.

## Data Availability

All data generated or analysed during this study are included in this published article.
